# Nonoperative Management for Traumatic Pseudoaneurysm of the Extrahepatic Portal Vein in an Older Adult

**DOI:** 10.31662/jmaj.2024-0089

**Published:** 2024-10-08

**Authors:** Chihaya Izumi, Junya Tsurukiri, Jushi Numata, Takeo Nagura, Hidefumi Sano

**Affiliations:** 1 Emergency and Critical Care Medicine, Tokyo Medical University Hachioji Medical Center, Tokyo, Japan

**Keywords:** multiple trauma, hepatic injury, portal vein

A 74-year-old man with hemorrhagic shock after a traffic accident was transferred to our emergency department. Contrast-enhanced computed tomography revealed intracranial and thoracic injuries (Figure 1), and the portal venous (PV) phase revealed a pseudoaneurysm of the superior mesenteric-PV (SMPV) confluence. Subsequently, the patient underwent nonoperative treatment with permissive hypotension. The traumatic PV pseudoaneurysm (tPVP) resolved three days posttreatment, and 20 days later, the diameter normalized (Figure 2).

**Figure 1.**
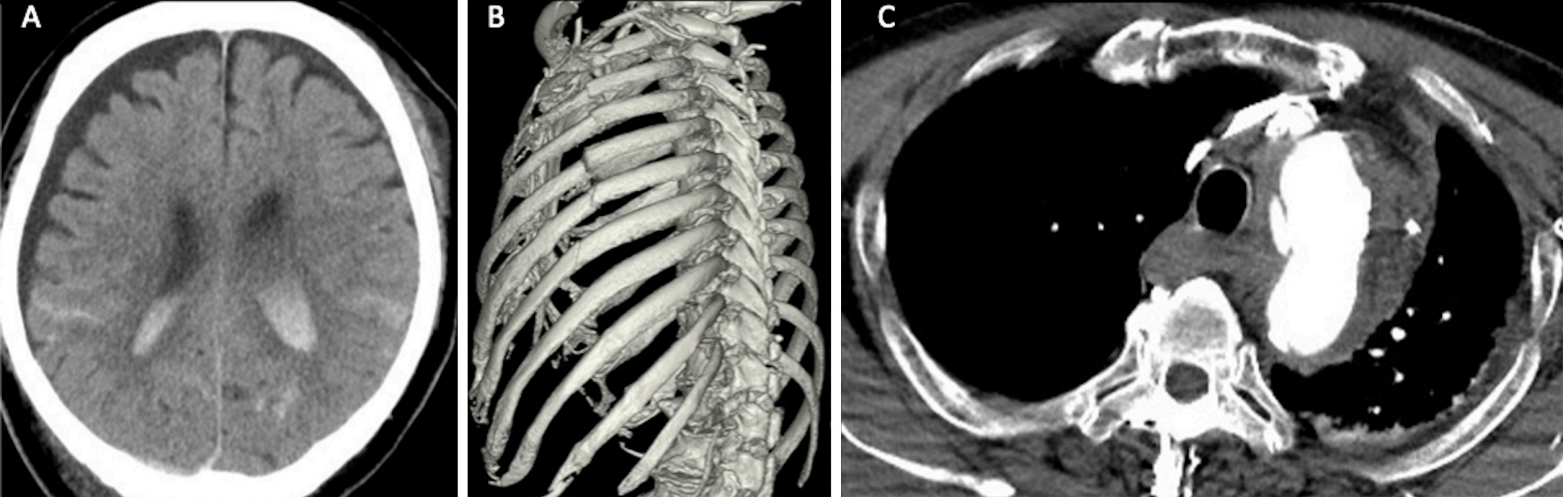
Computed tomography revealed intracranial injuries (A), multiple rib fractures (B), and aortic arch pseudoaneurysm (C).

**Figure 2.**
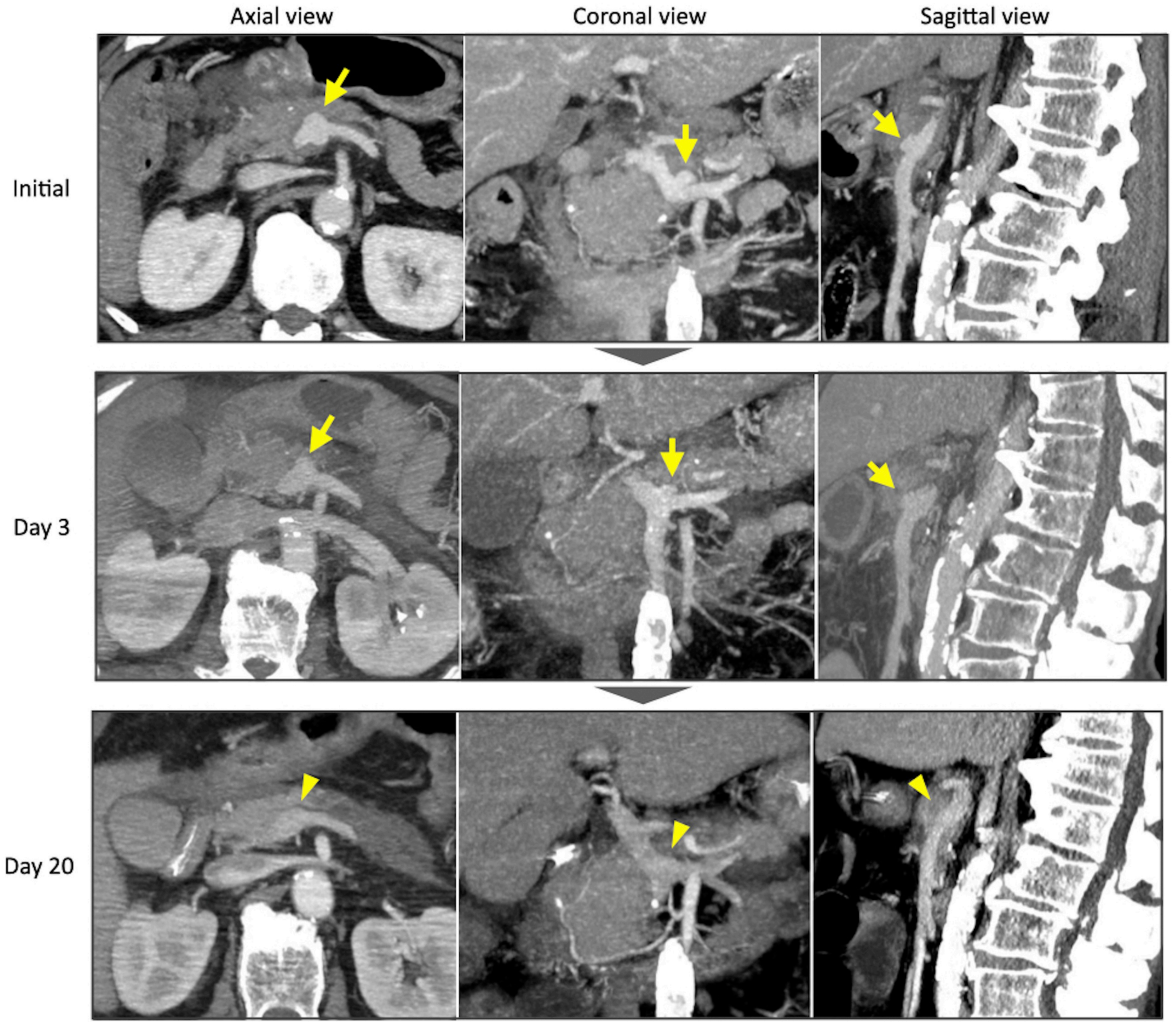
Progress images of portal vein pseudoaneurysm (arrow) detected by maximum intensity protection images from the portal venous phase of contrast-enhanced computed tomography. The pseudoaneurysm diameter normalized 20 days later (arrowhead).

tPV injury is a rare (~0.1% of cases) and lethal complication frequently affecting the portal triad ^(1), (2)^. Most tPVPs are caused by the shear force of the mobile mesentery, leading to avulsion of the SMPV confluence ^(1)^. Furthermore, tPVP is difficult to diagnose clinically and is usually identified intraoperatively, with surgical venorrhaphy/ligation being the standard treatment, which poses a high mortality risk ^(3), (4)^. Our treatment results suggest that PV blood flow reduction with permissive hypotension and careful imaging followup may prevent tPVP rupture nonoperatively.

## Article Information

### Conflicts of Interest

None

### Acknowledgement

The authors would like to thank Enago (www.enago.jp) for the English language review.

### Author Contributions

Conceived and designed the experiments: IC, TJ

Contributed to interpretation of data: NJ, NT

Approved the final version to be submitted: TJ, SH

### Informed Consent

Written informed consent was obtained from the patient for publication of this case report and any accompanying images.
